# The early response of renal cell carcinoma to tyrosine kinase inhibitors evaluated by FDG PET/CT was not influenced by metastatic organ

**DOI:** 10.1186/1471-2407-14-390

**Published:** 2014-06-02

**Authors:** Manabu Kakizoe, Masahiro Yao, Ukihide Tateishi, Ryogo Minamimoto, Daiki Ueno, Kazuhiro Namura, Kazuhide Makiyama, Narihiko Hayashi, Futoshi Sano, Takeshi Kishida, Kazuki Kobayashi, Sumio Noguchi, Ichiro Ikeda, Yoshiharu Ohgo, Masataka Taguri, Satoshi Morita, Tomio Inoue, Yoshinobu Kubota, Noboru Nakaigawa

**Affiliations:** 1 Department of Urology, Yokohama City University Graduate School of Medicine, 3-9 Fukuura Kanazawaku, Yokohama 236-0004, Japan; 2 Department of Radiology, Yokohama City University Graduate School of Medicine, Yokohama, Japan; 3 Department of Urology, Kanagawa Cancer Center, Yokohama, Japan; 4 Department of Urology, Yokosuka Kyosai Hospital, Yokosuka, Japan; 5 Department of Urology, Yokohama Minami Kyosai Hospital, Yokohama, Japan; 6 Department of Urology, Yokohama Sakae Kyosai Hospital, Yokohama, Japan; 7 Department of Biostatistics and Epidemiology, Yokohama City University Graduate School of Medicine, Yokohama, Japan; 8 Department of Biomedical Statistics and Bioinformatics, Kyoto University Graduate School of Medicine, Kyoto, Japan

**Keywords:** Renal cell carcinoma, Tyrosine kinase inhibitor, FDG PET/CT, Metastasis, Standardized uptake value, SUVmax, Organ

## Abstract

**Background:**

Tyrosine kinase inhibitors (TKIs) have become the mainstay of treatment for advanced renal cell carcinoma (RCC), but it has been unclear whether the antitumor effect of TKIs depends on the organ where the RCC metastasis is located. We previously reported that the FDG accumulation assessed by FDG PET/CT, was a powerful index for evaluating the biological response to TKI. In this study we investigated the differences in FDG accumulation and the response to TKI as assessed by FDG PET/CT among various organs where RCC were located.

**Methods:**

A total of 48 patients with advanced RCC treated with a TKI (25 with sunitinib and 23 with sorafenib) were evaluated by FDG PET/CT before and at 1 month after a TKI treatment initiation. The maximum standardized uptake value (SUVmax) of all RCC lesions were measured and analyzed.

**Results:**

We evaluated 190 RCC lesions. The pretreatment SUVmax values (mean ± SD) were as follows: in the 49 lung metastases, 4.1 ± 3.3; in the 40 bone metastases, 5.4 ± 1.6; in the 37 lymph node metastases, 6.7 ± 2.7; in the 29 abdominal parenchymal organ metastases, 6.6 ± 2.7; in the 26 muscle or soft tissue metastases, 4.4 ± 2.6; and in the nine primary lesions, 8.9 ± 3.9. Significant differences in the SUVmax were revealed between metastases and primary lesions (*p* = 0.006) and between lung metastases and non-lung metastases (*p* < 0.001). The SUVmax change ratios at 1 month after TKI treatment started were -14.2 ± 48.4% in the lung metastases, -10.4 ± 23.3% in the bone metastases, -9.3 ± 47.4% in the lymph node metastases, -24.5 ± 41.7% in the abdominal parenchymal organ metastases, -10.6 ± 47.4% in the muscle or soft tissue metastases, and -24.2 ± 18.3% in the primary lesions. There was no significant difference among the organs (*p* = 0.531).

**Conclusions:**

The decrease ratio of FDG accumulation of RCC lesions evaluated by PET/CT at 1 month after TKI treatment initiation was not influenced by the organs where the RCC metastasis was located. This result suggests that TKIs can be used to treat patients with advanced RCC regardless of the metastatic site.

## Background

Renal cell carcinomas (RCCs) account for 3% of all malignancies in adults [1]. Approximately 30% of RCC patients have metastases at the time of diagnosis, and 20%–40% of all patients relapse or develop metastases after radical nephrectomy with curative intent [2,3]. For many years, classical cytokine therapies had been the only systematic treatments available for advanced RCC, but the response rate to the cytokine therapies was only ~20% [4-6]. The development of novel and effective systematic therapeutics is desirable.

The oncogenic mechanism of RCC was recently elucidated, and agents have been developed that target the relevant biological pathway that has a critical and necessary role in RCC survival or progression. Tyrosine kinase inhibitors (TKIs) such as sunitinib and sorafenib, which target vascular endothelial growth factor (VEGF) receptors, improved the prognosis of patients with advanced RCC [7-9]. The antitumor activity of the TKIs was not cytotoxic, as are the classical antitumor therapeutics, but rather cytostatic, suppressing biological activity by inhibiting angiogenesis. Actually, some RCCs treated with TKIs did not decrease in tumor volume but entered a period of long-term dormancy, without an enlargement of volume or novel metastasis. A new biological marker evaluating the biological activities of RCC would be important if TKIs are to become the mainstay of treatment for advanced RCC.

Based on this concept, we have been investigating the utility of ^18^ F-2-fluoro-2-deoxyglucose positron emission tomography/computed tomography (FDG PET/CT), which is a useful non-invasive tool to evaluate glucose metabolic status, and reported the possibility of using the standardized uptake value (SUV), a semiquantitative simplified measurement of the tissue FDG accumulation rate, as a biomarker expressing the biological activity of RCC. We reported previously that the maximum SUV (which was the highest SUV in individual patients assessed by pretreatment FDG PET/CT) could predict survival [10]. We then found that the progression-free survivals of patients with RCC showing a ≥ 20% decrease in SUVmax at 1 month after TKI treatment started was longer than that of patients with RCC showing a < 20% decrease in SUVmax [11]. However, it has been unclear whether pretreatment FDG accumulation and its response to TKI were affected by the organs where the RCC metastases were located. In the present study we thus investigated the differences in FDG accumulation and its response to TKI among organs where RCC metastases were located.

## Methods

### Patients

We analyzed patients with advanced RCC pathologically diagnosed by prior nephrectomy or biopsy and treated by sunitinib or sorafenib between June 2008 and April 2013 at Yokohama City University hospital and its affiliated hospitals. The patients were initially assessed by conventional imaging techniques (computed tomography [CT], magnet resonance imaging [MRI], or bone scintigraphy) and diagnosed as stage IV or recurrent RCC. Patients with uncontrolled diabetes mellitus (blood glucose level >150 mg/dL) or with other known malignancies, and those treated with therapeutics during the 2 weeks prior to the scan were excluded. The study protocol was approved by the Yokohama City University Institutional Review Board. Written informed consent was obtained from all patients for enrollment in this study and publication of accompanying clinical records and images. The decision for patients to undergo therapy was made before the evaluation by FDG PET/CT.

### Treatment

Sunitinib was administered to each patient orally once a day at the dose of 50 mg in 6-wk cycles consisting of 4 wks of treatment followed by 2 wks without treatment. Oral sorafenib (800 mg) was administered daily. The dose of sunitinib was reduced to 37.5 or 25 mg and that of sorafenib was reduced to 600 or 400 mg according to the patient’s pretreatment general condition or major adverse events during treatment.

### Imaging

FDG PET/CT was performed in all patients before and 1 month after the TKI treatment started. Patients fasted for at least 6 h prior to an intravenous injection of ^18^F FDG. PET/CT images were obtained using a PET/CT system (Aquiduo 16; Toshiba Medical Systems, Tokyo). PET/CT images were acquired from the top of the head to the mid-thigh at 60 min after an intravenous injection of 2.5 MBq/kg of [^18^F] FDG. Mean of the time from injection to imaging was 60 min (standard deviation (SD) 6; range 50-84). Mean of FDG dosage was 151 MBq (SD 29; range 91-212). A low-dose non-contrasted CT scan was acquired first and used for attenuation correction. Emission images were acquired in 3-dimensional mode for 2 min per bed position. After PET acquisition, contrast-enhanced CT was performed with a 2-mm slice thickness, 120 kV, 400 mA, 0.5 s/tube rotation, from the top of the head to the mid-thigh, with breath holding. A total of 100 mL of contrast medium (iopamidol) was administered intravenously at a rate of 1.0 mL/s. The scan delay was set at 120 s after starting the injection of contrast material. The patients with a serum creatinine level >1.5 mg/dL were examined without contrast material. Images were reconstructed by attenuation-weighted ordered-subset expectation maximization (OSEM) (four iterations, fourteen subsets, 128 × 128 matrix, with 5-mm Gaussian smoothing). The standardized uptake value (SUV) was determined according to the standard formula, with activity in the volume of interest (VOI) recorded as Bq per mL/injected dose in Bq per total body weight (kg). The maximum SUV (SUVmax) was recorded using the maximum pixel activity within the VOI. Tumor size responses were evaluated by the Response Evaluation Criteria in Solid Tumors (RECIST) version 1.1. The VOI was settled to encompass the targets within areas of increased uptake and measured on each slice by two experienced physician, DU and KM who were blinded to clinical data. Discrepancies were resolved by consensus reading. Analysis of FDG uptake in the primary tumor was made with reference to contrast-enhanced CT images to differentiate tumor from physiologic parenchymal and urinary tract.

### Statistical analysis

The Kruskal-Wallis test and the Mann-Whitney *U*-test were used to assess the differences in pretreatment SUVmax, post-treatment SUVmax, SUVmax change ratio, and the diameter change ratio among organs where RCC metastases were located. In the assessment of the SUVmax change ratio, the RCC metastases which pretreatment FDG accumulations were not detected were excluded. In the assessment of diameter change ratio, the RCC metastases which diameter could not be measured were excluded. All statistical analyses were carried out with SPSS software (SPSS, Chicago, IL). Significance was assigned at *p* < 0.05.

## Results

### Patient characteristics and intervention

A total of 48 patients treated with TKIs (25 with sunitinib and 23 with sorafenib) were analyzed retrospectively. The clinical characteristics of the 48 patients are detailed in Table 1. There were 40 men and 8 women. The median age was 66 years (range 32 to 80). Of the 48 patients, 41 had clear cell carcinoma, five had papillary carcinoma, one had mixed clear cell and papillary renal carcinoma (a hemodialysis patient), and one had clear cell carcinoma mixed with a sarcomatoid component. There were 21 patients with recurrent diseases and 27 with stage IV disease.

**Table 1:** Patients and disease characteristics (n=48)

**Age (year)**	
32–80	(median 66)
**Gender**	
Male	40
Female	8
**Histology**	
Clear cell	41
Papillary	5
HD*	1
Clear/sarcomatoid	1
**MSKCC classification**	
Favorable	17
Intermediate	24
Poor	7
**Nephrectomy**	
Yes	38
No	10
**Prior systematic treatment**	
Non	30
Cytokine	14
TKI	4
**Evaluated RCC lesions**	(total 190)
Lung	49
Bone	40
Lymph node	37
Abdominal organ	29
Muscle/Soft tissue	26
Primary	9

HD*: mixed clear cell and papillary renal carcinoma in a hemodialysis patient.

According to the Memorial Sloan-Kettering Cancer Center (MSKCC) classification [12], 17 patients were classified as favorable risk, 24 as intermediate risk, and 7 as poor risk. Thirty-eight patients had undergone nephrectomy. Thirty patients had had no previous systematic therapies, and 18 patients had previous systematic therapies (one with sunitinib, one with sorafenib, two with sorafenib and interferon-alpha, 10 with interferon alpha, and 2 with interferon alpha and interleukin-2, 2 with chemotherapy). These treatments ended more than 2 wks prior to the pretreatment evaluation by FDG PET/CT.

In the 48 patients, we analyzed 190 RCC lesions diagnosed as RCC tumors by conventional imaging techniques. The numbers of RCC lesions in individual patients ranged from one to 10 (median three). There were 49 lung metastases (25.8%), 40 bone metastases (21.0%), 37 lymph node metastases (19.5%), 29 abdominal organ metastases including liver, adrenal gland, pancreas, spleen, contralateral kidney, uterus, and vagina (15.3%), 26 muscle or soft tissue metastases (13.7%), and nine renal primary sites (4.7%).

### The assessment by FDG PET/CT

**Figure 1:**
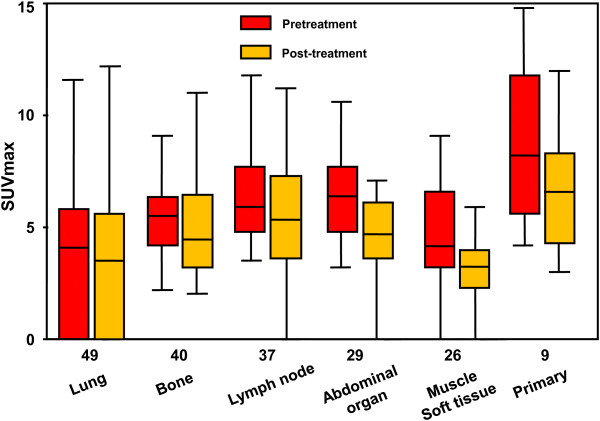
Pretreatment and post-treatment SUVmax values of the RCC patients.

We first analyzed the SUVmax values obtained by FDG PET/CT before treatment. The mean of interval between The SUVmax of all lesions was 5.5 ± 3.0 (mean ± SD). When the differences among organs were examined, we found that the pretreatment SUVmax values were: lung metastases 4.1 ± 3.3, bone metastases 5.4 ± 1.6, lymph node metastases 6.7 ± 2.7, abdominal organ metastases 6.6 ± 2.7, muscle or soft tissue metastases 4.4 ± 2.6, and primary sites 8.9 ± 3.9 with a significant difference (*p* < 0.001, Kruskal-Wallis test) (Figure 1). The mean of pretreatment SUVmax in all metastases was 5.3 ± 2.9 and there was statistical difference in SUVmax between renal origin and metastases (*p* = 0.006, Mann-Whitney *U*-test). When we analyzed the differences among metastases, it was revealed that the lung metastases demonstrated significantly lower SUVmax values compared to the non-lung metastases (*p* < 0.001, Mann-Whitney *U*-test).

We next analyzed the SUVmax assessed by FDG PET/CT performed 1 month after the TKI treatment initiation (day 30 ± 6; range 14 to 47). The post-treatment SUVmax of all 190 lesions was 4.6 ± 2.9 (mean ± SD). The post-treatment SUVmax values were: lung metastases 3.5 ± 3.0 , bone metastases 4.9 ± 2.2, lymph node metastases 5.6 ± 3.1, abdominal organ metastases 4.6 ± 2.8, muscle or soft tissue metastases 3.6 ± 2.5, and primary sites 6.6 ± 3.0, with a significant difference (*p* = 0.001, Kruskal-Wallis test) (Figure 1). The mean of post-treatment SUVmax in all metastases was 4.5 ± 2.8, and there was a significant difference in SUVmax between primary sites and metastases (*p* = 0.034, Mann-Whitney *U*-test). When the differences among metastasis locations were analyzed, the lung metastases demonstrated significantly lower SUVmax values compared to the non-lung metastases (*p* = 0.014, Mann-Whitney *U*-test).

**Figure 2:**
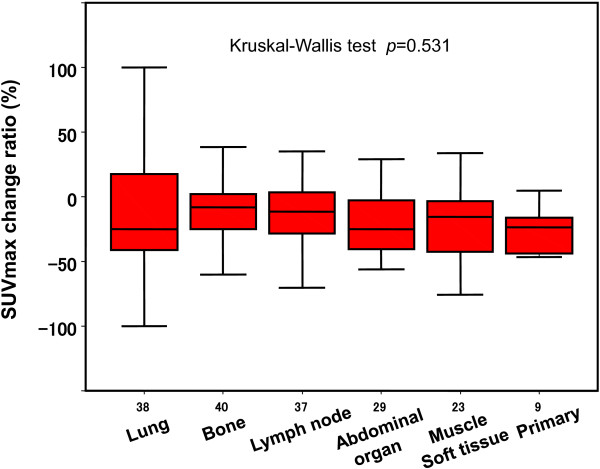
SUVmax change ratio after TKI treatment of the RCC patients.

The SUVmax change ratio of all 190 lesions was -14.1 ± 41.1% (mean ± SD). When we analyzed the differences among organs, we found that the SUVmax change ratio of the lung metastases was -14.2 ± 48.4%; that of bone metastases was -10.4 ± 23.3%, lymph node metastases -9.3 ± 47.4%, abdominal organ metastases -24.5 ± 41.7%, muscle or soft tissue metastases -10.6 ± 47.4%, and primary sites -24.2 ± 18.3%. There was no significant difference among all organs at which RCC metastases were located (*p* = 0.531, Kruskal-Wallis test) (Figure 2).

**Figure 3:**
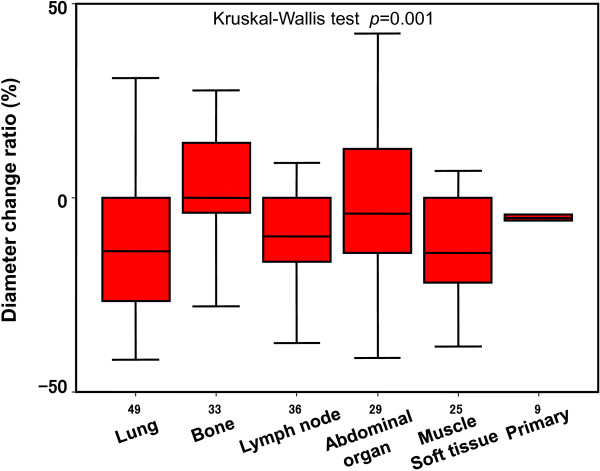
Diameter change ratio after TKI treatment of the RCC patients.

**Figure 4:**
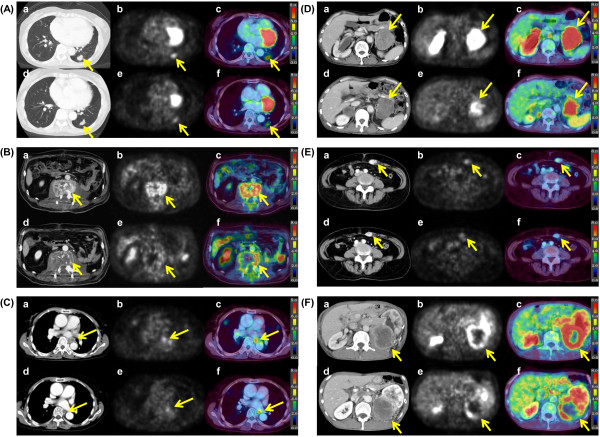
**FDG PET/CT features in the pretreatment state and post-treatment state.** CT imaging **(a)**, PET imaging **(b)** and fused imaging **(c)** before treatment and CT imaging **(d)**, PET imaging **(e)** and fused imaging **(f)** at1 month after TKI treatment initiation. **A**: A 55-year-old female with lung metastasis. She was treated with sorafenib. The SUVmax showed a 25.6% decrease (from 4.3 to 3.2) and the tumor dia. showed a 13.6% decrease. **B**: A 66-year-old male with lumbar vertebrae metastasis. He was treated with sunitinib. The SUVmax showed a 27.5% decrease (from 8.0 to 5.8) and the tumor dia. showed a 3.9% decrease. **C**: A 76-year-old male with mediastinal lymph node metastasis. He was treated with sorafenib. The SUVmax showed a 13.2% decrease (5.3 to 4.6) and the dia. showed a 10.0% decrease. **D**: A 64-year-old male with adrenal gland metastasis. He was treated with sunitinib. The SUVmax showed a 25.0% decrease (8.8 to 6.6) and the dia. a 8.5% decrease. **E**: A 67-year-old female with rectus abdominis muscle metastasis. She was treated with sorafenib. The SUVmax showed a 26.5% decrease (3.4 to 2.5) and the dia. a 16.0% decrease. **F**: A 71-year-old male with primary tumor in the left kidney. He was treated with sunitinib. The SUVmax showed a 21.4% decrease (8.4 to 6.6) and the dia. did not change.

Lastly, we investigated the change ratio of tumor diameter between before and after the start of TKI treatment. The ratio of all lesions was -5.0 ± 25.5% (mean ± SD). The diameter change ratio of the lung metastases was -9.2 ± 27.8%, that of the bone metastases 4.2 ± 15.4%, lymph node metastases -5.4 ± 36.4%, abdominal organ metastases -2.3 ± 21.2%, muscle or soft tissue metastases -11.3 ± 18.1%, and primary sites was -5.8 ± 7.2%. There was a significant difference among metastasis sites (*p* = 0.001, Kruskal-Wallis test) (Figure 3). There was a significant difference in the change ratio of tumor diameter between the bone metastases and non-bone metastases (*p* < 0.001, Mann-Whitney *U*-test).Figure 4 shows the FDG PET/CT features in the pretreatment state and post-treatment state of the lung metastases, bone metastases, lymph node metastases, abdominal organ metastases, muscle metastases, and primary tumors.

## Discussion

We and some other investigators have found that FDG PET/CT is a powerful tool to assess the biological status of RCCs, and we suggested the potency of FDG PET/CT as an imaging biomarker for advanced RCC [10,11,13-16]. One of the uses of an imaging biomarker is to evaluate individual lesions in a single patient. Gerlinger et al. reported that the gene-expression signature of RCC showed heterogeneity, for example between metastases and the primary lesion in an individual patient [17]. Their findings suggested that the biological evaluation of individual metastases of RCC in a single patient was important, but at the same time their report raised many clinical questions. One of the questions was whether the biological status of an RCC and its response to treatment are influenced by the organ at which the RCC metastasis is located. Here we investigated the differences among organs where RCC metastases were located using the assessments by FDG PET/CT.

We first focused on the differences in pretreatment FDG accumulation among the organ where RCC metastases were located. The primary origin showed higher pretreatment FDG accumulation compared to the metastases. Additionally, the lung metastases showed lower accumulation compared to non-lung metastases when the difference in FDG accumulation among only metastases was examined, although there was not statistical difference in the diameter between the lung metastases and non-lung metastases (data not shown). The present results support our hypothesis that the FDG accumulation expressed biological activity of RCC and RCCs showing high FDG accumulation predict a short survival time. Because, there are several clinical reports that the prognosis of RCC patients with primary tumors was poor compared with that of patients who had undergone a nephrectomy [18-20]. Additionally, lung metastases showed the better response to various therapies than non-lung metastasis and RCC patients with lung metastasis only show longer survival than other RCC patients [21-24]. In our study, there were only 2 patients with only lung metastasis. The difference of prognosis cannot be analyzed in such small sized study. The further study targeting large number of patients is necessary.Next, we focused on the change of FDG accumulation at 1 month after TKI treatment started, and we found that the change ratio of FDG accumulation was not significantly different among the organs where RCC metastases were located. However, the change ratio of SUVmax in the 190 lesions evaluated in this study showed a wide range (median -16%, range -100 to 137). An interesting finding of the present study was that the change ratio of FDG accumulation of the bone metastases was not significantly different from that of the none-bone metastases, although the sizes of the bone metastases did not reduce like that of the non-bone metastases did, as shown in Figures 2 and 3. Actually, the patient whose case is shown in Figure 4B, with bone metastasis showing a 28% decrease in the SUVmax after sunitinib treatment started, is alive with disease on the last observation, 40 months after the treatment started. These results indicate that the delivery and effect of TKIs are not influenced by the organ location of RCC metastasis, and they suggest that TKIs could be applied for patients with advanced RCC regardless of the metastatic site.

To our knowledge, this is the first report to analyze the differences in FDG accumulation of RCC among metastases locations. However, the number of patients was limited. A further study with a larger number of patients is necessary to test these results.

## Conclusion

The decrease ratio of FDG accumulation of RCC lesions evaluated by FDG PET/CT at 1 month after TKI treatment initiation was not influenced by the organs where the RCC metastasis was located. This result suggests that TKIs can be used to treat patients with advanced RCC regardless of the metastatic site.

## Competing interests

The authors declare that they have no competing interests.

## Authors’ contributions

Noboru Nakaigawa had full access to all of the data in the study and takes responsibility for the integrity of the data and the accuracy of the data analysis. All authors read and approved the final manuscript. Study concept and design: MK, MY, UT, RM, TI, YK, NN. Acquisition of data: KM, NH, FS, TK, KK, SN, II, YO. Analysis and interpretation of data: DU, KN, MK, MT, SM, NN. Administrative, technical, or material support: TI, YK, NN. Drafting of the manuscript: MK. Critical revision of the manuscript for important intellectual content: NN. Obtaining funding and supervision: NN.

## Pre-publication history

The pre-publication history for this paper can be accessed here:

http://www.biomedcentral.com/1471-2407/14/390/prepub
